# The impact of avoidable mortality on life expectancy at birth in Spain: changes between three periods, from 1987 to 2001

**DOI:** 10.1136/jech.2007.066027

**Published:** 2008-08-13

**Authors:** R Gispert, I Serra, M A Barés, X Puig, A Puigdefàbregas, A Freitas

**Affiliations:** 1 Servei d’Informació i Estudis, Departament de Salut, Generalitat de Catalunya, Barcelona, Spain; 2 Departament d’Estadística, ETSEIB, Universitat Politècnica de Catalunya, Barcelona, Spain

## Abstract

**Objective:**

To evaluate the impact of avoidable mortality on the changes in life expectancy at birth in Spain.

**Methods:**

Standard life table techniques and the Arriaga method were used to calculate and to decompose life expectancy (LE) changes by age, effects and groups of causes of avoidable mortality among three periods (1987–91, 1992–6 and 1997–2001). A list of causes of avoidable mortality reached by consensus and previously published in Spain was used.

**Main results:**

Life expectancy increased in all ages and both sexes. The main contribution to the increase of LE at birth was due to people over 50. Mortality in young adults produced a reduction in LE between the first two periods, but there was an important increase in LE between the last two periods; in both cases, this was the result of factors amenable to health policy interventions. The highest improvement in LE was due to non-avoidable causes, but avoidable mortality through health service interventions showed improvements in LE in those younger than 1 year and in those aged 45–75 years.

**Conclusions:**

Making a distinction between several groups of causes of avoidable mortality and using decomposition by causes, ages and effects allowed us to better explain the impact of avoidable mortality on the LE of the whole population and gave a new dimension to this indicator that could be very useful in public health.

Avoidable mortality has been proposed as an indicator to monitor health service results^1^ ^2^ and to compare the performance of different health systems. In the field of public health and health services research, much work has been previously published^3^ ^4^ although its use in practice has not yet become generalised. In Spain, this indicator was introduced at the beginning of the 1990s under the acronym MIPSE (Unnecessarily Premature and Amenable to Health Services Mortality),^5^ and an atlas with the distribution of avoidable mortality in autonomous communities was published^6^ that has been used in several studies and health reports.^7^ ^8^

The indicator of avoidable mortality is based on a selection of deaths that, according to the cause of death and age, should not have occurred because health measures exist that should have been sufficiently effective to avoid them.^1^ ^2^ Empirical studies analysing this indicator in different countries and over different time periods show consistent results which are compatible with the concept on which the indicator is based.^4^ In other words, time trends of avoidable mortality are more favourable than those of mortality that is considered to be non-avoidable or general. The geographical distribution of avoidable mortality is related to differences in socioeconomic status, the level of development of the country and, especially, the development of a country’s health system.^3^ ^4^ Some authors have distinguished between subgroups of causes of avoidable mortality to indicate the effects of different kinds of health interventions: primary or secondary prevention or treatment.^3^ ^9^

Within the limitations, the most important is the variability among studies of the different causes of deaths that are considered to be avoidable^4^ ^10^. A recent study showed that geographical variation and differences in time trends of avoidable mortality are very highly dependent on which causes of death are considered to be avoidable, as this can produce contradictory or inconsistent results.^11^ To counteract this problem and to make studies in Spain more consistent, a group of experts has updated the avoidable mortality list through a consensus process.^12^ Using this new list, the performance of the Spanish health system within autonomous communities has been analysed.^8^ The results showed that in the last 15 years avoidable mortality has decreased more than non-avoidable mortality, and particularly so for those causes of death that are influenced by the direct intervention of the healthcare system.^13^ In contrast, those causes of death that are influenced mainly by the policy measures taken in other sectors, which nevertheless have an impact on health, showed fluctuating results (initially there was an increase but this was followed by a decrease in deaths).^13^ Furthermore, the geographical pattern is also different for both groups of causes of death, with avoidable mortality due to causes influenced by healthcare being higher in the south of Spain and avoidable mortality due to causes influenced by health policy interventions being higher in regions along the Mediterranean coast.^13^

The objective of this work was to evaluate the impact of avoidable mortality on the health status of the general population by analysing the effect that avoidable mortality has had on the evolution of life expectancy (LE) at birth in Spain.

## METHODS

Deaths of residents in Spain from 1987 to 2001 and estimations of the inter-census populations on 1 July each year from 1987 to 2001 were used.^14^

The analysis was carried out for groups of causes, according to the list reached by consensus and previously published in Spain^12^ (table 1). The groups were: global avoidable mortality including all deaths on the list; avoidable mortality through health service interventions; avoidable mortality through intersectoral health policy interventions; and the remaining causes of death, which we called non-avoidable. The consensus process involved experts in medical specialties and public health and followed several criteria to update the already published lists of avoidable mortality. The most important were the availability in Spain of effective measures to treat or prevent the listed diseases; the increase in the upper age limit to fit with the important increase in life expectancy experienced; and that accidents and violence were considered to be preventable, which is how they are considered in the public health field.^12^

**Table 1:** Groups of causes of avoidable mortality in the Spanish consensus list (from Gispert *et al*.^12^)

Condition	Age	ICD-9 codes		ICD-10 codes
*Mortality avoidable by health service interventions*				
Tuberculosis (sequelae included)	0–74	010–018,137	A15–A19, B90	
Cervical cancer	15–74	180	C53	
Cancer of corpus uteri and cancer of unspecified part of uterus	15–74	182,179	C54, C55	
Hodgkin’s disease	0–74	201	C81	
Chronic rheumatic heart diseases	0–74	393–398	I05–I09	
Pneumonia, acute respiratory infections and influenza	0–74	480–486, 460–466, 487	A48.1, J12–J18 (except J18.2), J00–J06 (except J02.0, J03.0), J20–J22, J10–J11	
Asthma	5–49	493	J45–J46	
Diseases of the appendix	0–74	540–543	K35–K38	
Abdominal hernia	0–74	550–553	K40–K46	
Cholelithiasis/cholecystitis	0–74	574–575	K80–K82	
Hypertensive diseases	0–74	401–405	I10–I15	
Cerebrovascular diseases	0–74	430–438	I60–I69, G45, F01.1	
Maternal mortality (pregnancy, childbirth and puerperal complications)	All ages	630–676	O00–O99, A34	
Perinatal conditions	All ages	760–779	P00–P96, A33	
Female breast cancer	0–74	174	C50 (female)	
Ischaemic heart diseases	35–74	410–414	I20–I25	
Peptic ulcer	0–74	531–534	K25–K28	
Immunisation preventable	0–74	032, 037, 033, 055, 056, 072, 045, 070.0, 070.1, 070.2–70.3	A36, A35, A37, A49.2, B05, B06, B26, A80, B15, B16, B17.0, B18.0–B18.1	
Nutritional anaemias	0–74	280–281	D50–D53	
Cancer of skin (melanoma and non-melanoma)	0–74	172, 173	C43, C44, C46.0, C46.9	
Cancer of testis	0–74	186	C62	
Leukaemia	<15	204–208	C91–C95	
Disorders of the thyroid gland	0–74	240–246	E00–E07	
Diabetes mellitus	0–49	250	E10–E14	
Hyperplasia of the prostate	0–74	600	N40	
Congenital malformations of the circulatory system	0–74	745–747	Q20–Q28, I51.0	
Adverse events during surgical and medical procedures	All ages	E870–879	Y60–Y84	
*Mortality avoidable by health policy interventions*				
Cancer of trachea, bronchus and lung	0–74	162	C33, C34	
Alcoholic liver disease	15–74	571.0–571.3	K70.0, K70.1, K70.2–K70.3, K70.4, K70.9	
AIDS and HIV disease	All ages	279.5, 042, 279.6, 795.8*	B20–24, R75	
Traffic accident involving a motor vehicle	All ages	E810–825	V02–V04, V09 (except V09.1 and V09.9), V12–V14, V19.0–V19.2, V19.4–V19.6, V20–V79, V80.3–V80.5, V81.0–V81.1, V82.0–V82.1, V83–V88 (except V88.9), V89 (except V89.1)	
Suicide	All ages	E950–959	X60–X84, Y87.0	
Homicide	All ages	E960–969	X85–Y09, Y87.1	
Remainder of external causes (excludes traffic accidents involving a motor vehicle, suicide, homicide and iatrogenic causes)	All ages	E800–807E826–849E850–858E860–869E880–949E970–999	V01, V05–V06, V09.1, V09.9, V10–V11, V15–V18, V19.3, V19.8–V19.9, V80.0–V80.2, V80.6–V80.9, V81.2–V81.9, V82.2–V82.9, V88.9, V89.1, V90–V99, W00–W99, X00–X49, X50–X59, Y10–Y59, Y85–Y86, Y87.2, Y88–Y89, F10.0	

*When this disease was first recognised, different codes were used in several regions of Spain.

ICD, International Classification of Diseases.

Life expectancy at birth has been calculated according to standard life table techniques, with 85 and older being the last age group. The impact of avoidable mortality on the change of life expectancy at birth between time periods was estimated by decomposing life expectancy. Decomposition of differences in life expectancy was undertaken using the method developed by Arriaga^15^ with free software (Epidat 3.0) developed by the Regional Ministry of Health in Galicia, Spain, and the Pan American Health Organization.^16^

This method enables the separation of differences in life expectancy into factors related to age and cause of death, or to age and effects, expressed in years gained or lost. There are three additive effects:

- *direct effect*: years of life gained in the age group *x*, *x*+n owing to the mortality changes in the group itself
- *indirect effect*: years of life gained after the age *x*+n because of an increased number of survivors (as a result of the mortality changes of the age group *x*, *x*+n, assuming that the mortality of the older age groups does not change)
- *interaction*: years of life gained because of the supplementary survivors at age *x*+n as a consequence of mortality changes between *x* and *x*+n, taking into account that the mortality in the higher age groups also changes.

The open-ended age group (85 and over) is influenced only by a direct effect. The main difference between a direct effect and the other effects is that a direct effect measures the effect on LE that mortality changes in one age group produce in the group itself. Indirect and interaction effects measure the differences on LE that the mortality changes in one group produce through other age groups. See the supplementary material for further information on the calculations that were carried out.

The data were grouped into three periods of 5 years (P1, 1987–91; P2, 1992–6; P3, 1997–2001) to increase their consistency. The periods were chosen to fit as far as possible the LE trends experienced in Spain. Life tables for each one of the periods and for each sex were computed. For each change between two periods, the contribution of the groups of causes of mortality and of the effects by age and sex on the LE differences were analysed.

## RESULTS

The total number of avoidable deaths analysed was 1 023 036, which is 20% of the total mortality in the whole period (10.96% due to causes affected by health service interventions and 9.04% to causes avoidable by health policy actions). Non-avoidable causes accounted for the largest number of deaths and increased proportionally from one period to another (P1, 78.7%; P2, 79.42%; P3, 81.74%), whereas deaths due to avoidable causes decreased (P1, 21.3%; P2, 20.58%; P3, 18.25%) in relation to the total deaths in each period. There was a higher number of avoidable deaths in men than in women (25.86% versus 13.49%).

**Figure 1:**
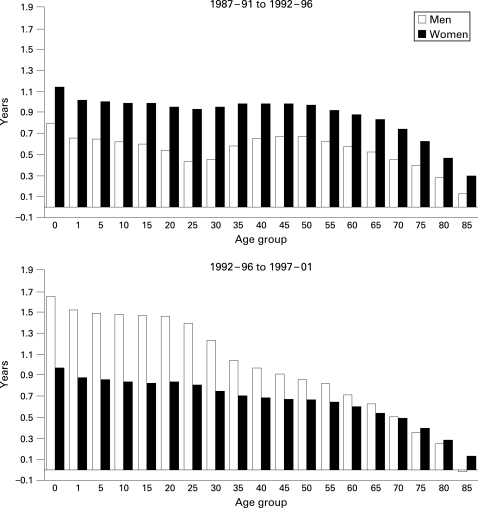
Differences in life expectancy by sex and age between the periods P1 and P2 and between the periods P2 and P3; Spain 1987–2001.

Figure 1 shows the changes in life expectancy, in number of years, by age and for both sexes between the analysed periods. Between the two first periods (P1 and P2) there is a greater increase in life expectancy at birth and at all ages in women. Men aged from 25 to 34 show the smallest increase. Between the next two periods (P2 and P3) the increase is higher in men, about 1.5 years, up to the age of 24 years, and in women the difference is around 1 year. In the older than 50 age group, the increase in life expectancy is similar in both sexes.

Table 2 shows the contribution of the three different effects to the changes in LE at birth, according to age group. Although LE was calculated in 5-year age groups with an open-ended group for age 85 and over, the results are presented for wider age groups to facilitate legibility. In both sexes the contribution to the increase of LE at birth in the age groups over 50 is notable. Furthermore, between periods P1 and P2 the mortality of 25–49-year-old men produced a reduction in LE, but between periods P2 and P3 there was an important increase in LE for men. With regard to the three different effects, for all ages and in both sexes, the most important contribution is due to the indirect effect. Moreover, the reduction in LE in young men within the first and second periods is shown for all of the effects. Direct effects are observed in all groups, but they are much more important after the age of 50.

**Table 2:** Contribution of the three different effects to the differences in life expectancy at birth by age and time period; Spain 1987–2001

Period	Effect	Males									
		0–24		25–49		50–84		⩾85		Total	
		N	%	N	%	N	%	N	%	N	%
1987–91 to 1992–6	Direct effect	0.0114	3.10	−0.0113	5.90	0.1124	19.09	0.0286	100	0.1411	17.80
	Indirect effect	0.3527	96.03	−0.1776	92.74	0.4605	78.24	–	–	0.6356	80.17
	Interaction	0.0029	0.79	−0.0026	1.36	0.0158	2.68	–	–	0.0161	2.03
	Total	0.3673	100	−0.1916	100	0.5885	100	0.0286	100	0.7928	100
1992–96 to 1997–2001	Direct effect	0.0076	2.66	0.0347	5.96	0.1441	18.25	−0.0053	100	0.1811	10.96
	Indirect effect	0.2722	95.11	0.5334	91.59	0.6258	79.26	–	–	1.4314	86.60
	Interaction	0.0062	2.17	0.0143	2.45	0.0198	2.50	–	–	0.0403	2.44
	Total	0.2862	100	0.5824	100	0.7895	100	−0.0053	100	1.6528	100

Period	Effect	Females									
		0–24		25–49		50–84		⩾85		Total	
		N	%	N	%	N	%	N	%	N	%
1987–91 to 1992–6	Direct effect	0.0048	2.20	0.0000	0.00	0.1470	18.32	0.1264	100	0.2782	24.45
	Indirect effect	0.2103	96.51	−0.0089	101.14	0.6254	77.93	–	–	0.8268	72.65
	Interaction	0.0029	1.33	0.0001	−1.14	0.0300	3.74	–	–	0.0330	2.90
	Total	0.2179	100	−0.0088	100	0.8025	100	0.1264	100	1.1380	100
1992–96 to 1997–2001	Direct effect	0.0035	2.00	0.0081	5.14	0.1032	17.93	0.0577	100	0.1725	17.86
	Indirect effect	0.1697	96.92	0.1471	93.40	0.4600	80.00	–	–	0.7768	80.41
	Interaction	0.0019	1.09	0.0023	1.46	0.0125	2.17	–	–	0.0167	1.73
	Total	0.1751	100	0.1575	100	0.5757	100	0.0577	100	0.9660	100

N, total number of years due to the corresponding effect in each age group.

Direct effect, years of life gained in the age group *x*, *x*+n owing to the mortality changes in the group itself.

Indirect effect, years of life gained after the age *x*+n because of an increased number of survivors (as a result of the mortality changes of the age group *x*, *x*+n, assuming that the mortality of the older age groups does not change).

Interaction, years of life gained because of the supplementary survivors at age *x*+n as a consequence of mortality changes between *x* and *x*+n, taking into account that the mortality in the higher age groups also changes.

**Figure 2:**
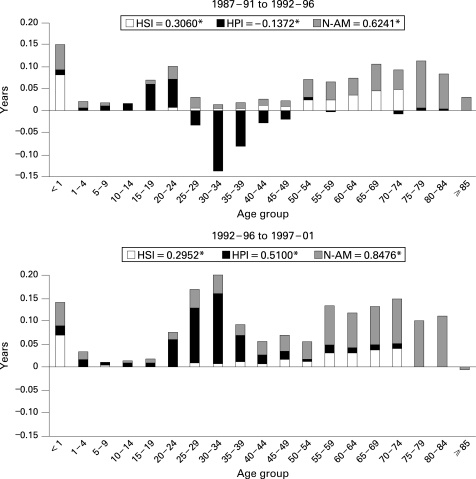
Contribution of causes and age to the differences in life expectancy at birth between time periods for men in Spain between 1987 and 2001. HSI, avoidable mortality by health service interventions; HPI, avoidable mortality by health policy interventions; N-AM, non-avoidable mortality. *The numbers are the total years of life expectancy gained due to each group of causes of death.

**Figure 3:**
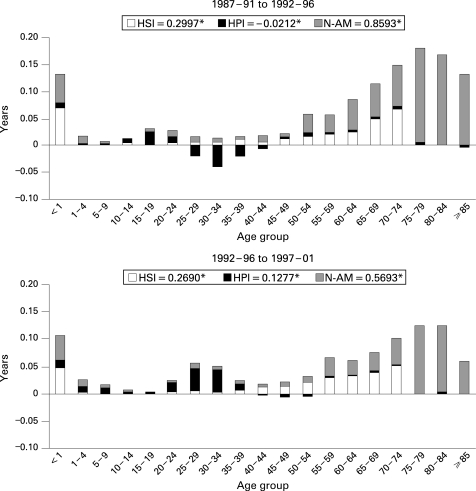
Contribution of causes and age to the differences in life expectancy at birth between time periods for women in Spain between 1987 and 2001. HIS, avoidable mortality by health service interventions; HPI, avoidable mortality by health policy interventions; N-AM, non-avoidable mortality. *The numbers are the total years of life expectancy gained due to each group of causes of death.

In figures 2 and 3 the contribution of causes and ages to the years of LE gained at birth, in number of years, is shown. It is evident that the highest number of years gained in both sexes is due to non-avoidable causes, mostly because of the contribution of ages older than 75, particularly in women, although in the last period the effect of avoidable causes is higher. The negative contribution of young people between the first two periods is basically a result of the mortality due to causes that can be affected by health policies in both sexes, but mainly in men. Nevertheless, between the second and third periods, these causes showed important gains in years of LE at birth for men. Finally, avoidable causes through health service interventions showed gains in LE in the groups younger than 1; the young and middle-aged population, especially in men; and the elderly, mainly in women.

## DISCUSSION

There has been an important increase in life expectancy at birth in Spain over the last 20 years, although during this time the improvement has not been consistent. The increase reflects the epidemiological profile of events affecting population health. For this reason it is very important to be able to identify the role that age and causes of death have played in this improvement, especially those causes that could be modified by available means.

Among the results, the negative impact on LE of causes amenable to health policies stands out, mainly in young age groups, between the first and second periods analysed. This is due to a combination of well-known factors that in recent years have threatened public health in Spain. There has been an increase in mortality for the younger age groups because of AIDS, drugs and traffic accidents,^17^ all of which are causes that are included in the group of avoidable mortality through intersector health policy interventions. The mortality from these causes was considerably reduced from the second to the third period as a result of the improvements in treatments for AIDS and a drastic reduction in the consumption of more lethal drugs and in traffic accidents.^18^ ^19^ As a result, these causes of death are among the highest contribution to the increase in LE.

Within the avoidable mortality through health services interventions group, a positive effect on LE stands out in the younger than 1 year age group as a consequence of the improvement in perinatal and neonatal mortality. Also in the age groups from 50 to 75 there is a positive effect because of a reduction in mortality due to cerebrovascular diseases, other heart diseases, respiratory and digestive diseases and female breast cancer, as shown in other studies on avoidable mortality.^4^ ^10^ ^20^ The improvement in mortality rates as a result of these diseases has been noted in different studies of mortality trends in Spain, and it has been attributed to a beneficial effect of the treatment and control of cardiovascular and respiratory risk factors and to the improvement in medical treatments.^17^ ^21^ ^22^

Nevertheless, the results also show an apparent paradox because non-avoidable causes of death seem to be producing the highest gains in LE. One explanation could be the significant contribution that non-avoidable causes make to the total number of deaths. However, if the results are viewed in relative terms, they show a different effect: a reduction of one-quarter of the total deaths produces around half of the years gained, basically in the second change of periods. As the group of non-avoidable causes results in the highest number of deaths, the reduction in mortality, despite being small, might contribute highly, in absolute terms, to the increase in LE.

Parallel results have been found in another study on avoidable mortality in Europe that had a similar methodology.^4^ That study pinpoints Spain as being different from the rest of Europe because of a reduction in LE in younger age groups that is attributed to other non-avoidable causes of mortality.^4^ The reasons for the differences observed with our results is that the European study included only those causes of avoidable mortality that are influenced by the intervention of health services, and also because the European study used temporary life tables (with an upper age limit of 75 years) for all causes. Our results show that this reduction in LE in young people is due to the causes that are sensitive to intersector policy interventions, and they reveal the usefulness of including this aspect of public health in the avoidable mortality list.

**What this study adds**

In Spain, the majority of the years of life gained or lost in young people are a consequence of the causes that are sensitive to intersector health policy interventions. Non-avoidable causes of death are those producing the largest increase in LE. As the group of non-avoidable causes results in the highest number of deaths, the reduction in mortality, despite being small, contributes highly to the increase in LE. By decomposing the changes in LE by effects it is shown that the majority of the years of life gained are due to an indirect effect. This means that the beneficial effect of the reduced mortality at one age is responsible for the years of life gained in other ages, and particularly in the LE of the whole population.

Another result to be highlighted is the one observed in the analysis of effects, which, we must remember, is a different interpretation of the same phenomenon: the decomposition of the changes in LE. The most important contribution to the increase in LE was due to the indirect effect in all ages. This means that a reduction in mortality in one age group has an impact in that group (as measured by the direct effect) and in other groups. Thus, the reduction in mortality as a result of particular causes that affect mainly specific age groups (eg AIDS or traffic accidents in young people) implies years of life gained by these young ages, and also by other age groups, and in this way it contributes to the increase in LE of the population as a whole. This could be a very important argument to support public health interventions, such as traffic regulations. The rationale is that mortality reductions in one group affect total life expectancy by means of other age groups.

In contrast, in older age groups, direct effect and non-avoidable causes play a more important role. This shows that a large proportion of the gains in LE for the whole of the population over recent decades has been produced by an improvement in survival for older people and this can explain, in part, the apparent paradox in the results mentioned above. In the definitions of avoidable mortality, most of the causes affected by health service interventions include death only before age 75, whereas after this age deaths are included in the non-avoidable group. Thus, longer survival in older people would have been the result of better health status (direct effect means LE gains in these ages) and of better medical treatment for avoidable causes, delaying death until after age 75. This result supports the theory about the usefulness of extending further the age limit used in the list of avoidable mortality.^4^

**Policy implications**

Differences in the quality of medical care and in health policy interventions among countries are being shown using the “avoidable mortality indicator”. The reduction in mortality due to these causes and its effect on the changes in LE at birth is a useful way to evaluate the impact of these interventions on population health. Life expectancy at birth has been increasing in recent decades owing to the reductions in mortality for middle-aged and older adults. Part of these changes is attributable to the contribution of medical care and another part to health policy interventions, which is a very important argument for public health purposes.

The impact of avoidable mortality on life expectancy has been previously analysed, with a different methodology, in one region of Spain.^23^ ^24^ The impact has also been analysed for other European countries with similar methods but using different avoidable mortality groups,^4^ ^9^ ^25^ ^26^ although some of these include causes related to health policies and causes related to health services.^9^ ^25^ ^26^ Unfortunately, the detailed lists of causes of death and ages are not the same, so the results are not completely comparable. The problem of comparability has been highlighted previously,^3^ ^4^ ^10^ ^12^ and it is common to all studies that have been carried out with this indicator. This drawback should be solved by establishing a consensus on the lists of causes. Probably, these lists should be adapted to the health situation in each country, so that the indicator is valid in every context. Nevertheless, common minimums (in some causes and age groups) should be maintained so that they could be comparable on an international level. For this reason, our work provides some important features. On the one hand, it uses a list of causes established by consensus in Spain for which the criteria used have been made public and discussed among professionals in the field.^27^ ^28^ On the other hand, it shows that making a distinction between the causes of death to be tackled by the health system and those needing policy interventions from other sectors gives a new dimension to this indicator. Furthermore, the combined use of decomposition in causes, ages and effects allows us a better understanding of the impact of avoidable mortality in the changes in LE experienced by the whole of the population, which could be very useful in public health.
